# A Hierarchical Predictive Coding Model of Object Recognition in Natural Images

**DOI:** 10.1007/s12559-016-9445-1

**Published:** 2016-12-28

**Authors:** M. W. Spratling

**Affiliations:** Department of Informatics, King’s College London, Strand, London, WC2R 2LS UK

**Keywords:** Predictive coding, Neural networks, Object recognition, Implicit shape model, Deep neural networks, Sparse coding

## Abstract

Predictive coding has been proposed as a model of the hierarchical perceptual inference process performed in the cortex. However, results demonstrating that predictive coding is capable of performing the complex inference required to recognise objects in natural images have not previously been presented. This article proposes a hierarchical neural network based on predictive coding for performing visual object recognition. This network is applied to the tasks of categorising hand-written digits, identifying faces, and locating cars in images of street scenes. It is shown that image recognition can be performed with tolerance to position, illumination, size, partial occlusion, and within-category variation. The current results, therefore, provide the first practical demonstration that predictive coding (at least the particular implementation of predictive coding used here; the PC/BC-DIM algorithm) is capable of performing accurate visual object recognition.

## Introduction

Localising and identifying items in visual scenes is of fundamental importance for many activities carried out by humans and other species. To solve this complex computational task, the brain is required to perform perceptual inference in order to find the most likely causes of the visual input. This process of object recognition is believed to be performed by a hierarchy of cortical regions along the ventral occipitotemporal pathway [1–4].

Predictive coding (PC) is a highly influential theory of cortical information processing [5–11]. PC is specifically suited to performing perceptual inference. Furthermore, PC can be implemented as a hierarchical neural network. PC should thus be suited, both at the functional and neurophysiological levels, to simulating object recognition. However, to date, this has not been demonstrated explicitly. This article presents the first demonstration that PC can perform object recognition in natural images. Specifically, the current results show that a particular implementation of PC (the PC/BC-DIM) algorithm^1^ can locate cars in natural images of street scenes, identify individuals from their face, and can categorize numbers in images of hand-written digits.

Object recognition requires the brain to solve an inverse problem: one where the causes (the shapes, surface properties, and arrangements of objects) need to be inferred from the perceived outcome of the image formation process. Inverse problems are typically ill-posed, meaning that they have multiple solutions (or none at all). For example, different sets of objects arranged in different configurations and viewed under different lighting conditions could potentially give rise to the same image. Solving such an ill-posed problem requires additional constraints to be imposed in order to narrow down the number of possible solutions to the single, most likely, one. In other words, constraints are required to infer the most likely causes of the sensory data. Constraints on visual inference might come from many sources, including knowledge learnt from prior experience (such as typical lighting conditions and the shapes and sizes of common objects), the recent past (knowledge about recently perceived causes, and expectations about how these might change or stay the same), and the present (such as information from elsewhere in the image or from another sensory modality).

PC proposes a scheme for applying such constraints in order to solve the inverse problem of vision. Specifically, PC suggests that the brain learns, from prior experience, an internal model of the world, or multiple models of specific aspects of the world embedded in different cortical regions. This internal model encodes possible causes of sensory inputs as parameters of a generative model (the weights of prediction neurons). New sensory inputs are then represented in terms of these known causes (by the activation of the prediction neurons). Determining which combination of the many possible causes best fits the current sensory data is achieved through an iterative process of minimising the error between the sensory data and the expected sensory inputs predicted by the causes. This inference process performs “explaining away” [14–18]: possible causes compete to explain the sensory evidence, and those causes that are best supported by the evidence, explain away that evidence preventing it from supporting competing causes. This suppression of alternative explanations typically results in a sparse set of predicted causes.

Object recognition requires perceptual representations that are sufficiently selective for shape and appearance properties (to distinguish one individual or one object category from another) as well as being sufficiently tolerant to changes in shape and appearance caused by illumination, viewpoint, partial-occlusion, within category variation, and non-rigid deformations (to allow the same object or object category to be recognised under different viewing conditions) [3, 4, 19–21]. It is generally believed that such selectivity and tolerance is built up slowly along the ventral pathway [22–28]. Different mechanisms are required to learn more selective representations and to learn more tolerant representations [20, 29]. Hence, several existing models of object recognition consist of alternating layers of neurons that perform these two operations in order to form more specialized representations in one layer, and more invariant representations in the next layer [20, 30–41].

The experiments described in this article were performed using a two-stage hierarchy of PC/BC-DIM networks. The same hierarchical arrangement of PC/BC-DIM networks has previously been used to model word recognition [42] (except this previous work, in contrast to the current work, used hard-coded weights and inter-stage feedback connections), and to model the learning of receptive fields in cortical areas V1 and V2 [18] (except that previous work used a different learning procedure to that described here). In the proposed model, the synaptic weights for alternate processing-stages are defined differently, in order to form receptive fields (RFs) that are specific to particular image features in one stage, and connections that generalize over these features in the subsequent stage. However, following learning, both stages operate identically. Both stages implement PC/BC-DIM, and hence, perform explaining away. The advantages of using explaining away to perform each of these operations have been demonstrated in two previous publications: [43] has shown that explaining away has advantages for producing neural responses that are selective to image features, while [44] has shown that explaining away has advantages for producing responses that generalise over changes in appearance. Here, it is shown that combining these two applications of PC/BC-DIM into one hierarchical neural network allows PC/BC-DIM to be used for object recognition.

## Methods

**Fig. 1:**
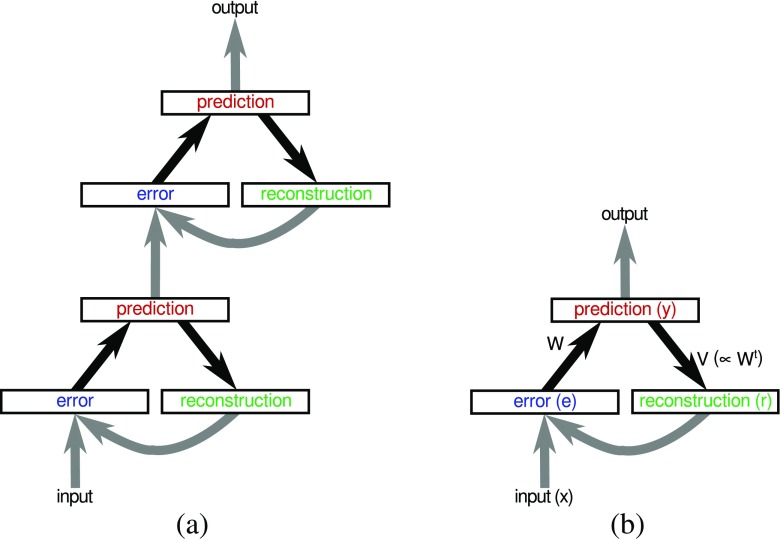
**a** The two-stage hierarchical PC/BC-DIM network used in the simulations described in this paper. *Rectangles* represent populations of neurons and *arrows* represent connections between those neural populations. The first processing-stage receives visual input. The second processing-stage receives input that is the steady-state prediction neuron responses generated by first processing-stage. **b** In each processing-stage, the population of prediction neurons constitute a model of the input environment of that processing-stage. Individual neurons represent distinct causes that can underlie the input (i.e., latent variables). The belief that each cause explains the current input is encoded in the activation level, *y*, and is used to reconstruct the expected input given the predicted causes. This reconstruction, *r*, is calculated using a linear generative model (see Eq. 1). Each column of the feedback weight matrix *V* represents an “elementary component,” “basis vector,” or “dictionary element,” and the reconstruction is thus a linear combination of those components. Each element of the reconstruction is compared to the corresponding element of the actual input, *x*, in order to calculate the residual error, *e*, between the predicted input and the actual input (see Eq. 2). The errors are subsequently used to update the predictions (via the feedforward weights *W*, see Eq. 3) in order to make them better able to account for the input, and hence, to reduce the error at subsequent iterations. The responses of the neurons in all three populations are updated iteratively to recursively calculate the values of *y*, *r*, and *e*. The weights *V* are the transpose of the weights *W* (but each set of weights may be normalised differently). Given that the *V* weights are proportional to the *W* weights, there is only one set of free parameters. All other connections (shown using gray arrows) are fixed to have binary values and to provide one-to-one connectivity between corresponding neurons in the pre- and post-synaptic populations

The experiments were performed using a two-stage hierarchical neural network model, as illustrated in Fig. 1a. The activations of the neurons in both stages were calculated using the PC/BC-DIM algorithm (as described in the “The PC/BC-DIM Algorithm” section). However, because different methods were used to learn the weights of each processing-stage (as described in the “Training” section), they played different roles in the object recognition process.

### Training

The training procedure for the first processing-stage was as follows.

#### **Image patches were extracted from the grayscale training images**

For those tasks in which the location and scale of the object was fixed (digit and face recognition), each training image was treated as a patch. In contrast, for those tasks in which the location of the object could vary (car recognition), patches were extracted from around keypoints (located using the Harris corner detector). Furthermore, in this case, to help distinguish cars (the “targets”) from other objects (the “non-targets”) that were also present in the test images, two sets of patches were obtained: those containing parts of the to-be-recognised objects, and those containing non-target image regions (obtained from images that did not contain the target object). To deal with changes in scale, the training images were rescaled to six different sizes, and patches were extracted from each set of resized training images.

#### **The image patches were clustered to form a dictionary**

The image patches were clustered using the hierarchical agglomerative clustering algorithm, with zero-mean normalized cross correlation (ZMNCC)^2^ between the most different members of each cluster as the measure of similarity. Clustering was terminated once the ZMNCC between all clusters was less than a similarity threshold (*κ*). Those clusters with fewer than *λ* members were discarded. The arithmetic mean of the patches forming the remaining clusters were used as the dictionary. For those tasks in which there were multiple classes (digit and face recognition), clustering was performed separately on the image patches extracted from images of each class. Similarly, for those tasks in which there was only one class of object to be recognized (cars), clustering was performed separately for target and non-target image patches. To deal with changes in scale, separate clustering of patches taken from each size of image was used.

The PC/BC-DIM algorithm can be used to allow the first processing-stage to find matches between the dictionary elements and an input image. The prediction neuron responses will represent the closeness of the match between the dictionary element and the image. If the dictionary elements are thought of as templates for object parts, then PC/BC-DIM can be considered as a method of template matching, but one that has considerable advantages over traditional template matching methods [43]. Specifically, by using PC/BC-DIM the match between a template and the image takes into account the evidence provided by the image and the full range of alternative explanations represented by the other templates. In other words, PC/BC-DIM performs explaining away. The result is that the prediction neuron responses (representing the match between templates and image locations) are very sparse. Those locations that match a template can therefore be readily identified and there is greater tolerance to changes in appearance due to changes in viewpoint [43].

Image features are better distinguished using relative intensity (or contrast) rather than absolute intensity. Hence, template matching was performed with the first processing-stage after the input image had been pre-processed as follows. The grayscale input image *I* was convolved with a 2D circular-symmetric Gaussian mask *g* with standard deviation equal to *σ* pixels, such that: $\bar {I}=I \ast g$. $\bar {I}$ is an estimate of the local mean intensity across the image. To avoid a poor estimate of $\bar {I}$ near the edges of the image, it was first padded on all sides by 4*σ* pixels with intensity values that were mirror reflections of the image pixel values near the edges of *I*. $\bar {I}$ was then cropped to be the same size as the original input image. The relative intensity can be approximated as $X=I-\bar {I}$. For biological-plausibility, the PC/BC-DIM algorithm requires inputs to be non-negative (weights and neural activations are also non-negative). To produce non-negative input to the PC/BC-DIM algorithm, the positive and rectified negative values of *X* (representing, respectively, increases and decreases in local contrast, or ON and OFF channels) were both used to form the input to the first processing-stage. The weights of each prediction neuron in the first processing-stage were defined by processing each dictionary element in an identical way to the input image. These weights were normalized so that the weights forming the RF of each prediction neuron summed to one.

The training procedure for the second processing-stage was as follows.

#### **First-stage prediction neuron responses were calculated for all the images in the training set**

The weights of the first processing-stage were defined as described in the preceding paragraph. An image from the training set (after being pre-processed as described in the preceding paragraph) was presented as input to the first processing-stage, and the PC/BC-DIM algorithm (as described in the “The PC/BC-DIM Algorithm” section) was executed. This was repeated for every image in the training set, and the first-stage prediction neuron responses to each training image were recorded.

#### **The second-stage weights were defined based on the responses of the first-stage prediction neurons**

A separate second-stage prediction neuron was defined to represent each object that was to be recognised. For those tasks in which the class or identity of the object was to be determined (digit and face recognition), a prediction neuron for each class or individual was defined. For tasks in which the location and scale of the object could vary (car recognition), prediction neurons were defined for each location and scale. The weights of these second-stage prediction neurons were set to be proportional to the sum of the responses of the first-stage prediction neurons to all training images containing the to-be-recognised object.

By having weights that connect a second-stage prediction neuron to all the prediction neurons in the first stage that represent (parts of) members of the to-be-recognized object category (at a specific scale or location), the second-stage prediction neuron will respond when those image features are identified by the first processing stage. The strength of response will depend not only on how many and how strongly the first processing stage templates match the image but will also depend on the weights of other second-stage prediction neurons. Specifically, the second processing stage performs explaining away, meaning that if an image feature is consistent with more than one of the objects represented by second-stage prediction neurons, then the PC/BC-DIM algorithm will activate the neuron corresponding to the most likely object and suppress the image feature’s support for alternative objects. The result is that the prediction neuron responses (representing the match between the image and a to-be-recognised objects) are very sparse. The true matches can therefore be readily identified and the generalisation over changes in appearance is more selective for those objects that have the most evidence [44].

For the task in which the location of the object could vary (i.e., car recognition), second-stage prediction neurons were defined to signal the presence of the object at each location. If the task had required the recognition of objects seen from different directions, or at different orientations, then it would have been necessary to define different second-stage prediction neurons to represent these different views of the same object. Such model neurons can be seen to be analogous to view-tuned cells observed in inferior temporal cortex [45, 46]. It would be possible to add a third processing stage to integrate information from such view-tuned neurons in order to signal the presence of the object irrespective of location or orientation. However, it is unlikely that such neurons, invariant to viewpoint, could be defined directly from the outputs of the first processing stage (i.e., by skipping the view-tuned neurons). This is because first-stage to view invariant connections would have to be very abundant, and this would allow the view invariant neurons to respond to combinations of image features that might appear in an image but not form the to-be-recognised object. In other words, attempting to increase tolerance to too quickly will lead to to a loss of selectivity. Hence, building PC/BC-DIM models that can recognise objects with greater tolerance to changes in appearance is likely to require the building of deeper hierarchical models [47, 48].

### Recognition

Following the training of both stages, described above, the hierarchical PC/BC-DIM model can be used to recognise objects in novel, test, images. The test image is pre-processed into ON and OFF channels as described in the “Training” section. These are input to the first processing stage, and the PC/BC-DIM algorithm (as described in the “The PC/BC-DIM Algorithm” section) is executed. The first-stage prediction neuron responses are then provided as inputs to the second processing stage and the PC/BC-DIM algorithm (as described in the “The PC/BC-DIM Algorithm” section) is executed for the second stage. The second-stage prediction neuron responses are then used to identify the to-be-recognised objects. For those tasks in which the location and scale of the object was fixed and for which each image contained exactly one object (digit and face recognition), the maximum response was taken to indicate the class of the image. For those tasks in which the location of the object could vary and in which the number of objects in each image could vary (car recognition), the presence of an object was indicated by prediction neurons responses that were peaks in the spatial neighbourhood and which exceeded a global threshold.

### The PC/BC-DIM Algorithm

The main mathematical operation required to implement the PC/BC-DIM algorithm is the calculation of sums of products. The algorithm can therefore be equally simply implemented using matrix multiplication or convolution.

The matrix-multiplication version of PC/BC-DIM is illustrated in Fig. 1b and was implemented using the following equations:

1 $$ r=Vy $$

2 $$ e=x \oslash \left[r\right]_{\epsilon_{2}} $$

3 $$ y \leftarrow \left[y \right]_{\epsilon_{1}} \odot We $$

Where *x* is a (*m* by 1) vector of input activations; *e* is a (*m* by 1) vector of error neuron activations; *r* is a (*m* by 1) vector of reconstruction neuron activations; *y* is a (*n* by 1) vector of prediction neuron activations; *W* is a (*n* by *m*) matrix of feedforward synaptic weight values, defined by the training process described in the “Training” section; *V* is a (*m* by *n*) matrix of feedback synaptic weight values; [*v*]_*𝜖*_ = max(*𝜖*, *v*); *𝜖*_1_ and *𝜖*_2_ are parameters; ⊘ and ⊙ indicate element-wise division and multiplication, respectively; and ← means that the left-hand side of the equation is assigned the value of the right-hand side. The matrix *V* is equal to the transpose of the *W* but each column of *V* is normalized to have a maximum value of one. Hence, the feedforward and feedback weights are simply rescaled versions of each other.

The convolutional version of PC/BC-DIM was implemented using the following equations:

4 $$ R_{i}= \sum\limits_{j=1}^{p} \left( v_{ji} \star Y_{j}\right) $$

5 $$ E_{i}=X_{i} \oslash \left[R_{i}\right]_{\epsilon_{2}} $$

6 $$ Y_{j} \leftarrow \left[Y_{j}\right]_{\epsilon_{1}} \odot \sum\limits_{i=1}^{k} \left( w_{ji} \star E_{i}\right) $$

Where *X*_*i*_ is a two-dimensional array representing channel *i* of the input; *R*_*i*_ is a two-dimensional array representing the network’s reconstruction of *X*_*i*_; *E*_*i*_ is a two-dimensional array representing the error between *X*_*i*_ and *R*_*i*_; *Y*_*j*_ is a two-dimensional array that represent the prediction neuron responses for a particular class, *j*, of prediction neuron; *w*_*ji*_ is a two-dimensional kernel representing the feedforward synaptic weights from a particular channel, *i*, of the input to a particular class, *j*, of prediction neuron, defined by the training process described in the “Training” section; *v*_*ji*_ is a two-dimensional kernel representing the feedback synaptic weights from a particular class, *j*, of prediction neuron to a particular channel, *i* of the input; and ⋆ represents cross-correlation. The weights *v*_*ij*_ are equal to the weights *w*_*ij*_ but are rotated by 180 ^∘^ and are normalised so that for each *j* the maximum weight value, across all *i*, is equal to one. Hence, the feedforward weights, between a pair of error-detecting and prediction neurons, and the feedback weights, between the corresponding pair of reconstruction and prediction neurons, are simply re-scaled versions of each other.

The matrix-multiplication and convolutional version of PC/BC-DIM are interchangeable, and which particular method was used depended on which was most convenient for the particular task. For example, the convolutional version was used when prediction neurons with identical RFs were required to be replicated at every pixel location in an image. To simplify the description of the proposed method, the rest of the text will refer only to the matrix-multiplication version of PC/BC-DIM.

For all the experiments described in this paper, *𝜖*_1_ and *𝜖*_2_ were given the values $\epsilon _{1}=\frac {\epsilon _{2}}{max\left (\tilde {V}\right )}$ (where $\tilde {V}$ is a vector containing the sum of each row of *V*, i.e., the sums of feedback weights targeting each reconstruction neuron) and *𝜖*_2_ = 1×10^−2^. Parameter *𝜖*_1_ prevents prediction neurons becoming permanently non-responsive. It also sets each prediction neuron’s baseline activity rate and controls the rate at which its activity increases when a new stimulus appears at the input to the network. Parameter *𝜖*_2_ prevents division-by zero errors and determines the minimum strength that an input is required to have in order to effect prediction neuron response. As in all previous work with PC/BC-DIM, these parameters have been given small values compared to typical values of *y* and *x*, and hence, have negligible effects on the steady-state activity of the network. To determine this steady-state activity, the values of *y* were all set to zero, and Eqs. 1 to 3 were then iteratively updated with the new values of *y* calculated by Eq. 3 substituted into Eqs. 1 and 3 to recursively calculate the neural activations. This process was terminated after 50 iterations. After 50 iterations, values of *y* less than 0.001 were set to zero. To perform simulations with a hierarchical model, the steady-state responses for the first processing-stage were determined. The first-stage prediction neuron responses were then provided as input to the second processing-stage, and Eqs. 1 to 3 applied to the second processing-stage to determine its response.^3^

The values of *y* represent predictions of the causes underlying the inputs to the network. The values of *r* represent the expected inputs given the predicted causes. The values of *e* represent the discrepancy (or residual error) between the reconstruction, *r*, and the actual input, *x*. The full range of possible causes that the network can represent are defined by the weights, *W* (and *V*). Each row of *W* (which correspond to the weights targeting an individual prediction neuron, i.e., its RF) can be thought of as a “dictionary element,” or “basis vector” or “elementary component” or “preferred stimulus,” and *W* as a whole can be thought of as a “dictionary” or “codebook” of possible representations, or a model of the external environment. The activation dynamics, described by Eqs. 1, 2, and 3, perform gradient descent on the reconstruction error in order to find prediction neuron activations that accurately reconstruct the input [14, 18, 62]. Specifically, the equations operate to find values for *y* that minimise the Kullback-Leibler (KL) divergence between the input (*x*) and the reconstruction of the input (*r*) [14, 63]. The activation dynamics thus result in the PC/BC-DIM algorithm selecting a subset of active prediction neurons whose RFs (which correspond to dictionary elements) best explain the underlying causes of the sensory input. The strength of activation reflects the strength with which each dictionary element is required to be present in order to accurately reconstruct the input. This strength of response also reflects the probability with which that dictionary element (the preferred stimulus of the active prediction neuron) is believed to be present, taking into account the evidence provided by the input signal and the full range of alternative explanations encoded in the RFs of the whole population of prediction neurons.

Compared to some earlier implementations of the PC/BC-DIM model, the algorithm described here differs in the following respects:

1. The calculation of the reconstruction error (in Eq. 2) is performed using max(*𝜖*_2_, *r*) rather than *𝜖*_2_ + *r*.
2. The calculation of the prediction neuron responses (in Eq. 3) uses max(*𝜖*_1_, *y*) rather than *𝜖*_1_ + *y*.
3. The value of *𝜖*_1_ is a function of the sum of the feedback weights targeting the reconstruction neurons rather than a fixed value (such as 1×10^−5^).

These changes help PC/BC-DIM to scale-up to very large networks of neurons. Specifically, for a very large population of prediction neurons, adding *𝜖*_1_ to each prediction neuron response (even when *𝜖*_1_ is very small) will cause the responses of the reconstruction neurons to be elevated, and the error neurons responses to be suppressed, which will in turn effect the prediction neuron responses. The second change above reduces this effect of *𝜖*_1_ on the neural responses. The first and third changes allow *𝜖*_1_ to be given the largest value possible (which speeds-up convergence to the steady-state) while preventing *𝜖*_1_ from effecting the responses.

In addition, in some earlier implementations of the PC/BC-DIM model, the reconstruction has been used purely as a means to calculate the errors, and hence, Eqs. 1 and 2 have been combined into a single equation. Here, the underlying mathematical model is identical to that used in previous work, but the interpretation has changed in order to consider the reconstruction to be represented by a separate neural population. This change, therefore, has no effect on the current results. However, other recent results have shown that a separate neural population encoding the reconstruction can perform a useful computational role [42, 64, 65].

### Code

Open-source software, written in MATLAB, which performs all the experiments described in this article is available for download from: http://www.corinet.org/mike/Code/pcbc_image_recognition.zip.

## Results and Discussion

### Handwritten Digit Recognition and Comparison with Deep Learning

**Fig. 2:**
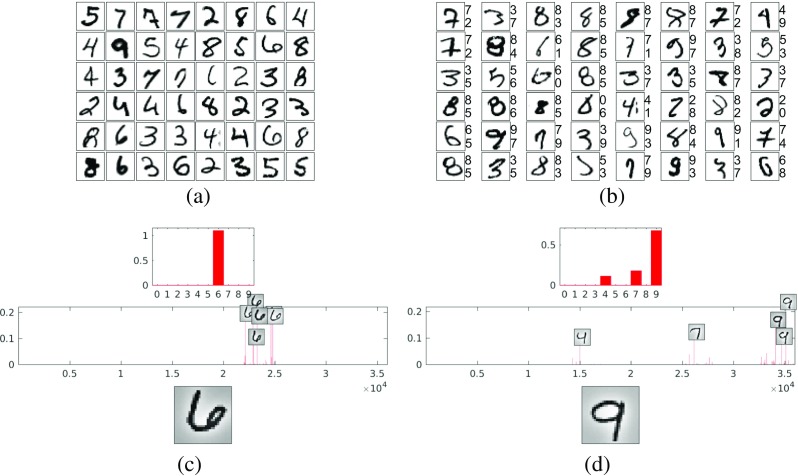
Results for the MNIST dataset. **a** Exemplars from the dictionary learnt from image patches. **b** Exemplars of misclassified images from the test set. There are two numbers to the *right* of each image. The *lower number* is the class predicted by the PC/BC-DIM network. The *top number* is the true class of the image. **c**, **d** show the responses of the prediction neurons to two images from the test set. Responses are shown as histograms where the *x*-axis is neuron number, and the *y*-axis is activation level (in arbitrary units). The *bottom panel* is the input to the PC/BC-DIM network. The *middle panel* shows the response of the prediction neurons in the first processing stage. The RFs of the most active prediction neurons are indicated by the images superimposed on the histogram. The *top panel* shows the response of the prediction neurons in the second processing stage

To test the ability of the proposed method to categorize images with tolerance to within-class variation, it was applied to the MNIST hand-written digits dataset.^4^ This datset consists of 28-by-28 pixel grayscale images of isolated digits. The training set contains 60,000 images and the test set contains 10,000 images. For this task, the following parameters were used: the similarity threshold for the clustering performed on the image patches was set equal to *κ* = 0.85; the threshold on the number of patches in each cluster was set equal to *λ* = 0; and the standard deviation of the Gaussian used to pre-process both the images and RFs of the first processing-stage was set equal to *σ* = 4 pixels. After pre-processing, each individual input image was rescaled to fill the range [0,1]. The training procedure for the first processing stage (see the “Training” section) produced a dictionary containing 35,956 elements. Examples of these dictionary elements are shown in Fig. 2a.

This dictionary was used to define the weights for 35,956 prediction neurons in the first processing stage (see the “Training” section). As there were ten classes, the second processing stage contained ten prediction neurons. The responses of the first- and second-stage prediction neurons to two test images are shown in Fig. 2c, d. When tested on all images from the test set, it was found that 2.19 % of these images were misclassified. Examples of incorrectly classified test images are shown in Fig. 2b. The classification error of the proposed method is compared to those of a variety of other algorithms in Table 1. It can be seen that while the results of the proposed method are good, they fall far short of the current state-of-the-art.

**Table 1:** Percentage classification error of various methods on the MNIST hand-written digits dataset

Method	MNIST
Hierarchical PC/BC-DIM	2.19
SVM [66]	12.0
MO-SFL [67]	6.55
ICA+ELM [68]	5.6
Spiking NN + unsupervised learning [69]	5.0
Spiking S2M + Event-driven CD [70]	4.4
PC/BC-DIM no pre-processing, classification via linear readout [71]	4.1
Nearest neighbour	2.77
Spiking DBN [72]	2.52
PC/BC-DIM no pre-processing, classification via sub-dictionary error [71]	2.19
Task-driven PSD [73]	1.98
DBN+SVM [66]	1.9
CNN (LeNet-1) [74]	1.7
Sprase coding [75]	1.26
DBN [54]	1.25
Stacked RBM [76]	1.2
Deep sparse rectifier neural network [77]	1.16
CNN (LeNet-4) [74]	1.1
SDL-G [78]	1.05
Deep Boltzmann machine [79]	0.95
CNN (LeNet-5) [74]	0.9
Sparse-HMAX+SVM (MTC) [80]	0.71
Locally shift invariant sparse hierarchical features [81]	0.64
Task-driven dictionary learning [82]	0.54
CNN (PSD) [40]	0.53
Multi-column deep neural network [83]	0.35
MCDNN [36]	0.23

Most of these state-of-the-art algorithms are deep hierarchical neural networks. Deep architectures can be sub-divided into two main types: (1) stacked generative models, such as deep belief networks [54, 55], and stacked autoencoders [56–58]; and (2) discriminative models with alternating layers of feature detection and pooling, such as convolutional neural networks CNN;[36–41], HMAX [20, 33–35, 61], and Neocognitron [30–32].

In common with architectures of the first type, the proposed algorithm also employs a hierarchy of generative models. However, the generative models are implemented using a different algorithm: PC/BC-DIM. Furthermore, PC/BC-DIM employs the generative model during inference: the generative model is used to make predictions of the expected sensory inputs, and through the iterative activation dynamics described by Eqs. 1 to 3, determine the prediction neuron activations that minimise the discrepancy between the predicted and actual inputs. In contrast, autoencoders and restricted Boltzmann machines RBM;[84, 85] which are the building blocks of previous architectures of the first type, only employ the generative model during learning. Once the weights have been set to allow these models to reconstruct the input, new inputs are processed using the feedforward weights only.

In common with architectures of the second type, the proposed algorithm has alternate processing stages that specialize in creating more discriminate representations in one layer, and more invariant representations in the next layer. This is achieved by defining the weights differently, but by applying the same algorithm to determine the neural activations during inference. In contrast, existing architectures of the second type use completely different mathematical operations to perform these two functions. For example, more specialized representations are often created by applying a linear filtering operation, while more tolerant representations are usually formed by finding the maximum response within a sub-population of pre-synaptic neurons. The proposed model is thus simpler, in that it only requires one type of processing stage.

Another difference between the proposed architecture and deep architectures of both type 1 and 2 is that in the proposed model, classification is performed by the last processing stage of the PC/BC-DIM hierarchy. In contrast, most existing deep architectures are used only as a method of feature extraction [57] to provide input to a distinct classification algorithm, such as a support vector machine (SVM) or a logistic regression classifier. The proposed model is thus simpler, in that it integrates feature extraction and classification within a single homogeneous framework, rather than using different methods for each.

However, as illustrated by the results in Table 1, deep architectures have an advantage in terms of classification accuracy. There are many reasons for this. Firstly, it is known that the deeper the architecture, the better the performance [86]. The proposed architecture is very shallow compared to most deep architectures. Creating deeper PC/BC-DIM hierarchies by stacking more processing-stages, might thus allow better performance, and potentially create a better model of the ventral pathway. However, doing so will require more sophisticated methods of defining the weights in those processing stages. The current model uses an unsupervized learning method. In contrast, much of the success deep architectures derives from using supervised learning. Using more training data is also known to generally improve performance. One way to generate additional training data is to generate images that are affine deformations of the original training images. This can result in a significant improvement in performance. For example, [83] report an error rate of 0.35 % on MNIST with deformation, and 1.47 % without.^5^ Expanding the dataset in this way could also be used to potentially improve the performance of the proposed PC/BC-DIM architecture. State-of-the-art performance on many classification tasks has been generated using an ensemble of deep architectures [36]: where multiple, different, deep networks are used to independently classify the input, and the final classification is a combination of these individual classifications. If classification accuracy, rather than biological-plausibility, were the main motivation then using the current architecture as the building block for an ensemble might also be considered.

### Face Recognition and Comparison with Sparse Coding

To test the ability of the proposed method to perform sub-ordinate level categorization (i.e., identification) with tolerance to illumination, it was applied to the cropped and aligned version of the Extended Yale Face Database B^6^ [87, 88]. This dataset consists of 168-by-192 pixel grayscale images of faces taken from a fixed viewpoint in front of the face under varying lighting conditions. There are approximately 64 images for each of 38 individuals. Following the method used in previous work with this dataset [89–93], half the images for each class were used for training and the other half for testing.

In previous work, classification has been performed using images down-sampled to 21-by-24 pixels (or fewer). This has been necessary as previous methods have used pre-processing steps (such as the calculation of Eigenfaces and Laplacian-faces) that are too memory intensive to be performed on larger images [89]. To allow a direct comparison with this previous work results are presented for the proposed method using images that have also been resized by a scale factor $\delta =\frac {1}{8}$ to 21-by-24. However, as the proposed method can work successfully with larger images, results are also presented for images at the original size (i.e., for *δ* = 1).

**Fig. 3:**
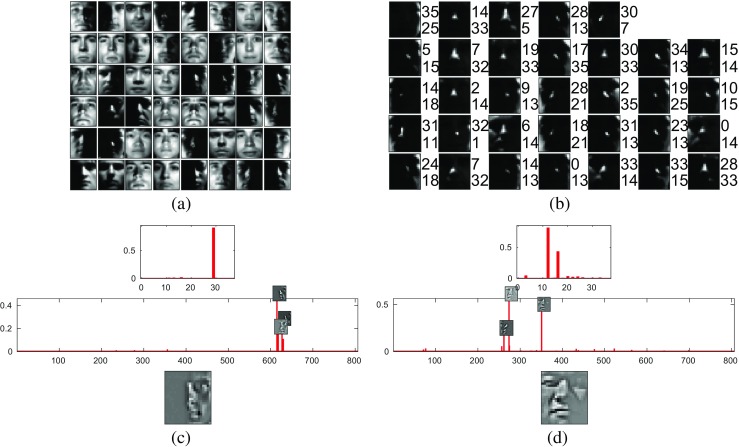
Results for the Extended Yale Face Database B, when using 21-by-24 pixel images. **a** Exemplars from the dictionary learnt from image patches. **b** All of misclassified images from the test set. There are two numbers to the right of each image. The *lower number* is the class predicted by the PC/BC-DIM network. The *top number* is the true class of the image. **c**, **d** show the responses of the prediction neurons to two images from the test set. The *bottom panel* is the input to the PC/BC-DIM network. The *middle panel* shows the response of the prediction neurons in the first processing stage. The RFs of the most active prediction neurons are indicated by the images superimposed on the histogram. The *top panel* shows the response of the prediction neurons in the second processing stage

For this task, the following parameters were used: the similarity threshold for the clustering performed on the image patches was set equal to *κ* = 0.9; the threshold on the number of patches in each cluster was set equal to *λ* = 0; and the standard deviation of the Gaussian used to pre-process both the images and the RFs of the first processing-stage was set equal to $\sigma =2.5\sqrt {\delta }$ pixels. After pre-processing, each individual input image was rescaled to fill the range [0,1]. For the 21-by-24 pixel images, the training procedure for the first processing stage (see “Training” section) produced a dictionary containing 806 elements. Examples of these dictionary elements are shown in Fig. 3a. This dictionary was used to define the weights for 806 prediction neurons in the first processing stage (see “Training” section). As there were 38 individuals, the second processing stage contained 38 prediction neurons. The responses of the first- and second-stage prediction neurons to two test images are shown in Fig. 3c, d. The incorrectly identified test images, for the 21-by-24 pixel version of this task, are shown in Fig. 3b. It can be seen that all the misclassified images were taken under very poor lighting conditions.

The classification error of the proposed method is compared to those of a variety of other algorithms in Table 2. It can be seen that the performance of the proposed method is competitive with the current state-of-the-art for this task. The current state-of-the-art algorithms are based on sparse coding. These algorithms represent the image using a sparse set of elements selected from an overcomplete dictionary. They then perform classification by analysing the reconstruction errors produced by dictionary elements associated with different classes [71, 75, 89, 93]. In common with these algorithms, PC/BC-DIM also represents the input images using a sparse code (examples can be seen in the lower histograms in Fig. 3c, d, where it can be seen that only a very small subset of the first stage prediction neurons are active). However, in contrast to most existing sparse dictionary-based classifiers, the proposed method makes the classification using the sparse code (the prediction neuron responses) rather than the reconstruction error (the error neuron responses). This latter method is more biologically-plausible, but less accurate [71]. It has been found that the performance of sparse dictionary-based classifiers is improved by the supervised learning of more discriminative dictionaries [75, 82, 92, 94–96]. Such learning might potentially also improve the performance of the proposed algorithm.

**Table 2:** Percentage classification error of various methods on the Extended Yale Face Database B

Method	YALE (21x24)	YALE (168x192)
Hierarchical PC/BC-DIM	2.7	0.5
Nearest neighbour [89]	9.3	
D-KSVD [93]	4.4	
LC-KSVD2 [91, 92]	3.3	
Laplacianfaces+SVM [89]	2.3	
SRC [89]	1.9	

### Car Recognition and Comparison with Generalized Hough Transform

To test the ability of the proposed method to localize and recognize objects in natural images with tolerance to position, illumination, size, partial occlusion, and within-category shape variation, it was applied to the UIUC cars dataset [97, 98].^7^ This dataset consists of greyscale images of outdoor scenes. The training set consists of 550 car images and 500 images that do not contain cars. There are two sub-tasks: recognising side views of cars at a single scale (the location and number of cars varies between test images), and recognizing side views of cars across multiple scales (the size, location, and number of cars varies between test images). For the single-scale task, the test set contains 170 images containing 200 side views of cars. The multi-scale task has a test set of 108 images containing 139 cars.

**Fig. 4:**
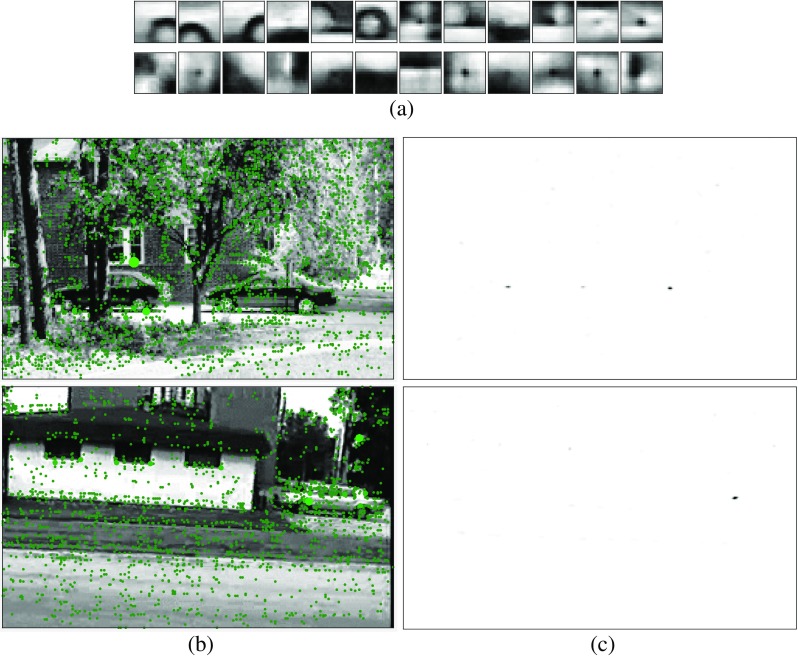
**a** A small sample of the dictionary elements represented by the first-stage prediction neurons. The *top row* shows RFs of prediction neurons trained on patches taken from the car images. The *second row* shows RFs of prediction neurons trained on patches taken from the non-car images. **b** Two example test images from the UIUC single-scale cars dataset [97, 98]. The *green dots* show the locations where dictionary elements representing car parts have been matched to the image: the size of the dot is proportional to the strength of the response of the corresponding first-stage prediction neuron. **c** The response of all the second-stage prediction neurons to the corresponding example test image shown in **b**. The response is indicated by the grayscale, with white corresponding to no response and black corresponding to a high response. It can be seen that the strongest responses correspond to the centres of the cars

The same training set, and the same parameter values, were used for both sub-tasks. Specifically, the similarity threshold for the clustering performed on the image patches was set equal to *κ* = 0.4, the threshold on the number of patches in each cluster was set equal to *λ* = 12, and the standard deviation of the Gaussian used to pre-process both the images and the RFs of the first processing stage was set equal to *σ* = 3.5 pixels. Training of the dictionary used to define the weights of the first processing stage was performed on 15-by-15 pixel patches extracted from the training images around keypoints located using the Harris corner detector. For the single-scale task, the patches taken from the car images were clustered into 273 dictionary elements. The non-car image patches were clustered into 140 dictionary elements. Examples of these first-stage dictionary elements are shown in Fig. 4a. These dictionary elements were used to define the RFs of the prediction neurons in the first PC/BC-DIM processing stage, resulting in 413 prediction neurons at each pixel location in the input image. For the multi-scale task, training was performed on the 1050 car and non-car training images resized to six different scales. The dictionary consisted of 2465 elements representing non-car parts and 3601 elements representing car parts, resulting in 6066 first-stage prediction neurons at each pixel location.

Figure 4b shows two example test images for the single-scale task on which have been superimposed dots to show locations where there is a strong response from the sub-population of first processing stage prediction neurons that represent car parts. The size of the dot is proportional to the magnitude of the response of the prediction neuron. For prediction neurons whose RFs were defined using the same dictionary element, non-maximum suppression was performed over those prediction neuron responses, so that all response other than the local maximum were set to zero.

For the single-scale task, the number of second-stage prediction neurons was equal to the number of pixels in the input image. Each second-stage prediction neuron had the same weights (but at spatially sifted positions), equal to the summed response of all the first-stage prediction neurons to all the car images in the training set. However, to improve tolerance to position, these weights were smoothed across space by convolving them with a two-dimensional circular symmetric Gaussian function with a standard deviation of two pixels. Figure 4c shows the responses of all the second-stage prediction neurons for the two images shown in Fig. 4b. For the multi-scale task, the second processing-stage consisted of six sub-populations of prediction neurons (one for each scale), each sub-population contained one prediction neuron for each pixel in the test image. In this case, the weights were smoothed across space and scale using a three-dimensional Gaussian function.

To determine the location of cars predicted by the proposed method, the spatial distribution of prediction neuron responses (as illustrated in Fig. 4c) was analyzed to find the coordinates of spatially contiguous regions of strong activity. Such a region was defined as a contiguous neighborhood in which each neuron had an activity of more than 0.001, and which was completely surrounded by neurons with a response of 0.001 or less. The coordinates represented by such a region were then determined using population vector decoding [99]. This simply calculates the average of the coordinates represented by the neurons in the region, weighted by each neuron’s response. For the multi-scale task, the coordinates of regions of high activity were determined in the same way, but in a three-dimensional space (position and scale). The total sum of the response in each region was also recorded.

To quantitatively assess the performance of the proposed algorithm, the procedures advocated in [98] were followed. Specifically, for each region with a total response exceeding a threshold, the location (and scale) represented by that region were determined (as described in the preceding paragraph) and these values were compared to the true location (and scale) of each car provided in the ground-truth data. The comparison was performed using the java code supplied with UIUC cars data set. If the predicted parameter values were sufficiently close to the ground-truth, this was counted as a true-positive. If multiple regions of high activity corresponded to the same ground-truth parameters, only one match was counted as a true-positive, and the rest were counted as false-positives. All other regions of high activity that failed to match the ground-truth data were also counted as false-positives. Ground-truth parameters for which there was no corresponding values found by the proposed method were counted as false-negatives. The total number of true-positives (TP), the number of false-positives (FP), and the number of false-negatives (FN) were recorded over all test images, and were used to calculate recall ($\frac {\text {TP}}{\text {TP}+\text {FN}}$) and precision ($\frac {\text {TP}}{\text {TP}+\text {FP}}$). By varying the threshold applied to select regions of high activity, precision-recall curves were plotted to show how detection accuracy varied with threshold. To summarize performance, the *f* score ($=\frac {2.\mathrm {recall.precision}}{\text {recall} + \text {precision}}=\frac {2\text {TP}}{2\text {TP}+\text {FP}+\text {FN}}$) which measures the trade-off between precision and recall, was calculated at the threshold that gave the highest value. In addition, to allow comparison with previously published results, the equal error rate (EER) was also found. This is the percentage error when the threshold is set such that the number of false-positives equals the number of false-negatives.

**Fig. 5:**
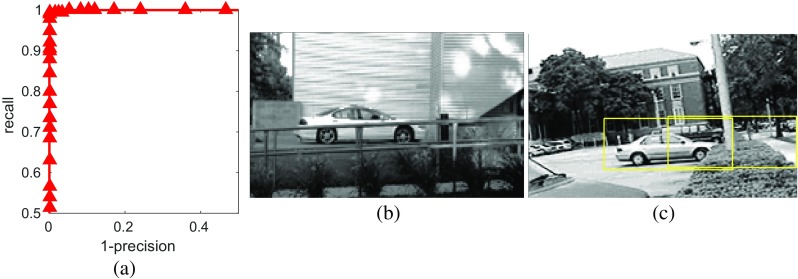
Results of applying the proposed method to the single-scale UIUC cars dataset. **a** Recall versus 1-precision. At the threshold for equal error rate, there were two images in which there were errors. **b** The only false negative. **c** The only false positive. The *bounding boxes*, shown in *yellow*, indicate locations in which cars were detected by the proposed algorithm

**Fig. 6:**
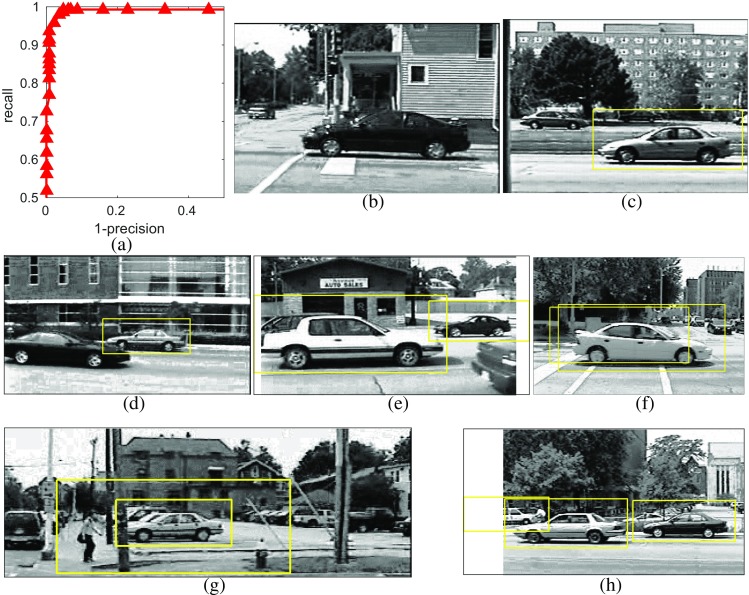
Results of applying the proposed method to the multi-scale UIUC cars dataset. **a** Recall versus 1-precision. At the threshold for equal error rate, there were seven images in which there were errors. These images are shown in (**b**–**h**) with *bounding boxes*, in *yellow*, indicating locations in which cars were detected by the proposed algorithm. **b**–**d** Shows the three images in which there were false negatives. **e** Shows the only image in which there was both a false negative and a false positive. Note that while both cars appear to have been recognized, one has not be located with sufficient accuracy. **f**–**h** Shows the three images in which there were false positives. Note that the last image has been flagged as containing a false-positive as the left-most car is not included as a true-positive in the ground-truth data

The precision recall curve obtained on the UIUC single-scale cars dataset is shown in Fig. 5. The *f* score was 0.9975 and the EER was 0.5 %. Figure 5b, c shows the only two images in the test set on which the proposed method makes a mistake at the threshold for equal error rate. The results obtained on the UIUC multi-scale cars dataset are shown in Fig. 6. In this case, the *f* score was 0.9718 and the EER was 2.9 %. These results are compared to those of other published methods in Table 3. It can be seen that the proposed method is competitive with the state-of-the art, and particularly, that it outperforms the method described in [44]. That method is similar to the one proposed here, except that the first processing-stage described here was replaced by a process that found keypoints in the image, and matched (using the ZMNCC as the similarity metric) the image patches around these keypoints to elements in the dictionary. Hence, the method proposed here is simpler, in that both stages are implemented using PC/BC-DIM, rather than being implemented in completely different ways.

**Table 3:** Percentage EER of various methods on the UIUC single-scale and multi-scale cars dataset

Method	UIUC-single	UIUC-multi
Hierarchical PC/BC-DIM	0.5	2.9
ISM [100]	9	–
ISM+MDL verification [100]	2.5	5
Hough Forest [101, 102]	1.5	2.4
Discriminative HT [103]	1.5	–
ESS [104]	1.5	1.4
Keypoint patch matching+PC/BC-DIM voting [44]	1	3.6
Chains model [105]	0.5	–
Sliding window HMAX+verification [106]	0.06	9.4
IHRF [107]	0	1.3
PRISM [108]	–	2.2

The algorithm described in [44] was inspired by the implicit shape model ISM;[100], which employs the generalised Hough transform [109–111] to allow dictionary elements that match features in the image to cast votes for the possible location and scale of the to-be-recognised object. Once all the votes have been cast, ISM uses a minimum description length (MDL) criteria to reject false peaks caused by votes that come from image elements which have also voted for other peaks that are more likely to be the true ones. The second processing stage in the proposed model can also be thought of as implementing the voting process of the generalized Hough transform, but using explaining away (rather than MDL) to suppress false peaks [44]. In a previous section, the function of the second processing stage was described as being analogous to the function of the pooling stages in deep neural networks. There is therefore also an analogy between the Hough transform and pooling. Both attempt to allow recognition with tolerance to location, but the Hough transform is both less constrained and less arbitrary than the pooling used in deep networks.

## Conclusions

The current work provides an initial proof-of-concept demonstration that predictive coding can perform object recognition in natural images. Hence, it provides concrete support for previous speculation about the possible role of predictive coding in perceptual inference. Object recognition is a complex task that requires being able to distinguish one individual or class of object from other individuals or classes while being able tolerate changes in the appearance of the to-be-recognised object from one image to another. The results presented here show that PC/BC-DIM can recognize individuals and classes, and that it can do so with tolerance to position, illumination, size, partial occlusion, and within-category shape variation. The experiments used here have not addressed tolerance to non-rigid shape deformations, or rotations.

As discussed in the “Results and Discussion” section, the proposed model has strong similarity to existing methods like deep neural networks, ISM, and sparse dictionary-based classification. These previous methods tend to make use of different mechanisms to perform different sub-tasks. For example, deep networks use different mechanisms for feature detection, pooling, and classification, while ISM uses different mechanisms for detecting image features and counting votes. In contrast, the proposed model uses the same mechanism (PC/BC-DIM) to perform each of these sub-tasks.

Improving the performance of the proposed method on the tasks used here, or extending it to more complex object recognition tasks that require tolerance to a greater range of image transformations of the recognition of a wider range of objects, or developing it into a model of ventral stream processing, is likely to require the building of deeper and more complex networks. Defining appropriate weights for such networks is the key to their success. In the current article, the weights have been set in a rather ad-hoc and non-biologically plausible way. This is sufficient for a proof-of-concept demonstration, but would need to be addressed in future work.

## Footnotes

1 PC/BC-DIM is a version of PC [7] reformulated to make it compatible with biased competition (BC) theories of cortical function [12, 13], and that is implemented using divisive input modulation [DIM; [14]] as the method for updating error and prediction neuron activations. DIM calculates reconstruction errors using division, which is in contrast to other implementations of PC that calculate reconstruction errors using subtraction [6, 10, 12]. The divisive method is preferred as it results in non-negative firing-rates and is thus more biologically-plausible [10, 12]. Furthermore, it has stable dynamics and converges more quickly allowing it to be used to build large-scale models [10, 14].

2 Also known as the sample Pearson correlation coefficient.

3 Determining, sequentially, the steady-state responses for each processing stage was necessary in order to make the proposed model tractable given the available computational resources (a Core i7-4790K desktop PC with 16GB RAM). A more biologically-plausible model would iterate Eqs. 1 to 3 for both processing stages simultaneously, with the prediction neuron response calculated for the first-stage at each iteration provided as input the second processing stage before the next iteration. In such an implementation, it would also be possible to explore the effects of inter-stage feedback connections from the second to the first processing stage. In the current, more tractable implementation, such connections would have no effect as the first stage has finished processing by the time the second stage starts. However, psychophysical experiments showing that image classification can be determined very rapidly in humans and monkeys [3, 49–53] suggest that cortical feedback connections (which would be modelled by inter-stage feedback) have little influence on object recognition (in unambiguous cases). The lack of inter-stage feedback connections in the current model also allows more direct comparison to other neural model of object recognition that contain only feedforward connections [e.g.,[20, 30–41, 44, 54–61]].

4
http://yann.lecun.com/exdb/mnist/

5
http://people.idsia.ch/∼ciresan/results.htm

6
http://vision.ucsd.edu/∼leekc/ExtYaleDatabase/ExtYaleB.html

7
https://cogcomp.cs.illinois.edu/Data/Car/
